# Appréciation de la sévérité de l'affection des patients admis en réanimation par la mesure de la CRP

**DOI:** 10.11604/pamj.2015.20.401.4311

**Published:** 2015-04-23

**Authors:** Jackson Djuma, Prosper Kalenga, Costa Kazadi, Diego Orbegozo, Jean Louis Vincent

**Affiliations:** 1 Service de Réanimation Médico-Chirurgicale, Cliniques Universitaires de Bruxelles, Belgique; 2 Département des Sciences Biomédicales, Cliniques Universitaires de Lubumbashi, République Démocratique du Congo

**Keywords:** Protéine C réactive, score APACHE, score SOFA, défaillance multiviscérale, C-reactive protein, APACHE score, SOFA score, multiple organ failure

## Abstract

**Introduction:**

La CRP est produite en grande quantité dans les processus inflammatoires aigus et chroniques. L'hypothèse de ce travail est que la CRP permet, à faible coût, d'apprécier la sévérité des patients admis en réanimation.

**Méthodes:**

La valeur journalière du taux sérique de CRP a été corrélée aux scores APACHE II à l'admission et SOFA pris quotidiennement durant le séjour en réanimation. La population étudiée était constituée de 100 patients admis en réanimation aux cliniques universitaires de Bruxelles.

**Résultats:**

Les patients septiques avaient un taux de CRP plus élevé comparé aux non septiques. Les patients ayant une CRP élevée avaient les scores APACHE (26 ± 6 vs 20 ± 9, p < 0,05), SOFA à l'admission (8 ± 4 vs 4 ± 3, p < 0.05), SOFA maximum plus élevés (9 ± 4 vs 7 ± 3 et 9 ± 4 vs 7 ± 3, p < 0.05) et un séjour en réanimation plus long. Les patients ayant un plus grand nombre d'organes défaillants ont la CRP la plus élevée (365 mg/L vs 80 mg/L, 205 mg/L vs 60 mg/L, 110 mg/L vs 60 mg/L, 150 mg/L vs 60 mg/L).

**Conclusion:**

Chez les patients présentant un taux sérique de CRP élevé, les scores de gravité (APACHE et SOFA), le pourcentage de patients présentant une infection et la durée du séjour en réanimation sont plus élevés.

## Introduction

La défaillance d'organes représente la première cause de décès en réanimation [1]. Cholongitas [2] suivi récemment par O'Brien [3] ont trouvé une mortalité de 90% chez les patients avec 3 organes ou plus défaillants. Le dosage de certaines protéines réactives de la phase aiguë a été proposé comme marqueur de sévérité de plusieurs pathologies, (procalcitonine dans les infections bactériennes, …), et comme reflet des atteintes tissulaires [4, 5]. La phase aiguë d'une réponse à l'agression stimule des mécanismes de défense et d'adaptation à l'agression tissulaire. Parmi ces marqueurs, nous pouvons citer la protéine C-réactive(CRP), la protéine amyloïde sérique A (SAA), les protéines du complément, le fibrinogène, l'alpha-1 antitrypsine, l'haptoglobine, l'antagoniste au récepteur à l'interleukine 1, l’‘hepcidine et la ferritine [6–7].

Ces protéines sont libérées en plus grande quantité dans les processus inflammatoires aigus et chroniques associés à l'infection, au traumatisme, à l'infarctus, aux inflammations articulaires et plusieurs cancers [5]. La CRP est une protéine produite par le foie suite à une stimulation par certaines cytokines telles que l'interleukine 6 et le tumornecrosis factor; elle est libérée dans la circulation 6 à 48h après cette stimulation [8].

Certaines études ont indiqué que la CRP peut être un marqueur de la défaillance d'organes [9–13] et est un bon marqueur de la mortalité chez les patients ayant une défaillance multi-viscérale (MOF) en réanimation [14]. Surtout la persistance de concentrations élevées de CRP est corrélée avec une augmentation du risque de défaillance d'organe et de décès [15]. Malgré le fait qu'en pratique, bien que suggéré par quelques observations isolées, l'intérêt clinique d'un suivi systématique de la CRP n'est pas démontré et son utilisation n'est pas recommandée par les experts [15] pour identifier l'infection, la CRP reste fort utilisée en réanimation aux cliniques universitaires de Bruxelles. Garde-t-elle encore sa valeur pronostique’ Une confirmation des résultats trouvés par Lobo [14] sur un échantillon moindre, aurait une valeur scientifique importante, bien que n'ayant pas de valeur statistique.

L'infection étant un phénomène microbien caractérisé par une réponse inflammatoire liée à la présence de microorganisme ou l´invasion de tissu de l'hôte normalement stérile par ces organismes [16].

L'hypothèse de ce travail est que la gravité des patients admis en soins intensifs est différente selon que le taux sérique de la CRP est inférieure à 10, comprise entre 10 et 100 et supérieure à 100.

L'objectif principal de ce travail sera de tester l'association entre le taux de CRP et la gravité des patients admis et hospitalisés en réanimation évaluée par les scores APACHE (Acute Physiology And ChronicHealth Evaluation) et SOFA (SequentialOrganFailureAssessment). Cela pour voir si elle permet toujours d'apprécier la sévérité de l'affection des patients admis en réanimation malgré sa forte utilisation dans ce service. Les objectifs secondaires dans ce travail seront d’établir une association entre d'une part la valeur de la CRP et d'autre part l'incidence de la défaillance d'organes, la durée du séjour en réanimation, la durée de jours sous hémofiltration, la prévalence de l'infection.

## Méthodes

Il s'agit d'une étude prospective observationnelle menée dans le Service des Soins Intensifs (35 lits) aux cliniques universitaires de Bruxelles. La population étudiée était constituée de 100 patients admis consécutivement en réanimation durant la période allant du 23 juillet 2012 au 6 septembre 2012. La valeur maximale de leur CRP a été prise journalièrement durant un séjour en soins intensifs de 7 jours. Ces 100 patients ont été ensuite catégorisés selon qu'ils avaient une valeur de CRP inférieure à 10, comprise entre 10 et 100 et supérieure à 100 mg/L. La CRP moyenne et médiane a été ensuite calculée selon ces 3 catégories.

Seuls les nouveaux cas admis à partir du 23 juillet 2012 ont été pris en compte. Sur 219 patients admis du 23 juillet 2012 au 6 septembre 2012, nous avons exclus 105 cas (48%) de chirurgie élective (surtout cardiovasculaire et neurochirurgicale), 9 réadmissions et 5 patients de moins de 18 ans. Ainsi 100 patients ont été retenus pour l’étude.

Pour la collecte des données, les valeurs les plus pathologiques ont été considérées dans ce travail: CRP maximale par 24 heures, valeurs les plus pathologiques des éléments constitutifs du score APACHE II et du score SOFA. Les patients ont été subdivisés en 3 groupes selon la valeur moyenne ou médiane de la CRP: < 10 mg/L (groupe 1), 10-100 mg/L (groupe 2), >100 mg/L (groupe 3). Nous avons calculé le score APACHE pendant les premières 24 heures après l'admission en réanimation, la CRP et le score SOFA ont été mesurés quotidiennement jusqu´au 7ème jour(J7) après l´admission ou jusqu’à la sortie du patient vivant ou mort. Nous avons considéré une défaillance d'organe pour un SOFA égale à 3 ou à 4. L'infection a été définie selon ACCP-SCCM Consensus Conference (1992). Nous n'avons pas fait de différence entre infection à l'admission ou infection acquise durant l'hospitalisation. Dans cette étude, nous avons considéré un patient comme ayant une infection si, à un moment donné de son séjour en réanimation, l’équipe médicale (staff) l'a considéré comme ayant une infection.

Les analyses statistiques ont été réalisées avec le programme statistique IBM SPSS Statistics 19 pour Windows (IBM Corporation, Somers, NY). Le test de Kolmogorov-Smirnov a été utilisé pour vérifier la normalité des distributions des variables continues. Les variables catégorielles sont exposées en nombre (n) et en pourcentage (%). Les variables continues sont exprimées en moyenne ± SD (l’écart-type) ou en médiane (percentile 25 - percentile 75). Pour comparer les caractéristiques démographiques et cliniques des groupes étudiés, le test de kruskal-Wallis, le test par ANOVA, le test-t, le test Mann-Whitney, le test Chi-Carré ou le test exact de Fisher sont utilisés. La correction de Bonferroni est utilisée dans le cas des comparaisons multiples. Les mesures répétées sont comparées à l'aide de la procédure « modèles mixtes linéaires ». Les valeurs de p < 0.05 sont considérées statistiquement significatives.

## Résultats

Les données démographiques de 100 patients retenus dans l’étude sont présentées dans le Tableau 1. Un tiers des patients étaient compris dans le groupe 1, un autre tiers groupe 2 et le dernier tiers groupe 3.

**Tableau 1:** Caractéristiques de base et de comorbidité des patients étudiés

Variables	Groupe 1 (CRP < 10 mg/L)	Groupe 2 (CRP 10-100 mg/L)	Groupe3 (CRP > 100 mg/L)
Patients, n (%)	35 (35)	32 (32)	33 (33)
Age (années)	57 ± 19	66 ± 14	60 ± 17
Hommes, n (%)	21 (60)	18 (56)	16 (48,5)
Score APACHE II	20 ± 9	24 ± 8	26 ± 6†
SOFA Amission	4 ± 3	6 ± 3	8 ± 4†
SOFA Max.	5 ± 3	7 ± 3	9 ± 4†‡
Séjour réa.	1 (0-3)	2 (1-4)	3 (2-7) †‡
Patients avec infection (%)	7 (20)	16 (50) †	24 (73) †
Patients en CVVH (%)	0 (0)	1 (3,1)	4 (12,1)
Urgences (%)	20 (57)	15 (47)	13 (39)
Etages (%)	11 (31)	13 (41)	11 (33)
Autre hôpital (%)	5 (14)	4 (12,5)	95 (27)
Cat. Médicale (%)	17 (49)	21 (66)	24 (73)
Chirurgie (24H) (%)	18 (51)	11 (34)	9 (27)
Neurochirurgie (%)	11 (31)	3 (9)	3 (9)
Traumatique (%)	6 (17)	7 (22)	2 (6)
Cardio vasculaire (%)	1 (3)	0 (0)	1 (3)
Abdominale (%)	0 (0)	1 (3)	3 (9)
Thoracique (%)	0 (0)	0 (0)	0 (0)
Diabète (%)	7 (20)	4 (12,5)	3 (9)
Cancer (%)	4 (11)	7 (22)	8 (24)
I.R.C. (%)	4 (11)	7 (22)	3 (9)
BPCO (%)	6 (17)	4 (12,5)	7 (21)
Cirrhose(%)	1 (3)	2 (6)	1 (3)
IC (NYHA III-IV) (%)	1 (3)	6 (19)	5 (15)
Cancer hémato (%)	0 (0)	1 (3)	0 (0)
Mortalité (%)	1 (17)	6 (19)	10 (30)

* Les résultats sont présentés sous forme de moyenne ± DS ou sous forme de pourcentage

† p < 0,05 vs groupe 1

‡ p < 0,05 vs groupe 2

Les patients ayant une CRP élevée ont un état plus grave, reflété par un score APACHE plus élevé (groupe 3 vs groupe 1, p < 0,05), un score SOFA à l'admission plus élevé (groupe 3 vs groupe 1, p < 0.05), un score SOFA maximum plus élevé (groupe 3 vs groupe 2 et groupe 3 vs groupe 1, p < 0.05), ont un séjour plus long en réanimation (groupe 3 vs groupe 2 et groupe 3 vs groupe1, p < 0.05), sont plus nombreux en hémofiltration (groupe 3 vs groupe 2 et groupe 3 vs groupe 1, NS). Les patients ayant une CRP > 100 mg/L ont une mortalité plus élevées comparée à celle des patients ayant une CRP< à 100 mg/L (groupe3 vs groupe 2 et groupe 3 vs groupe 1, NS). La prévalence des patients présentant une infection est également plus élevée chez les patients ayant un taux de CRP > 10mg/L comparé à ceux ayant un taux de CRP < 10 mg/L (groupe 2 vs groupe 1 et groupe 3 vs groupe 1, p < 0.05). (Tableau 1).

**Figure 1:**
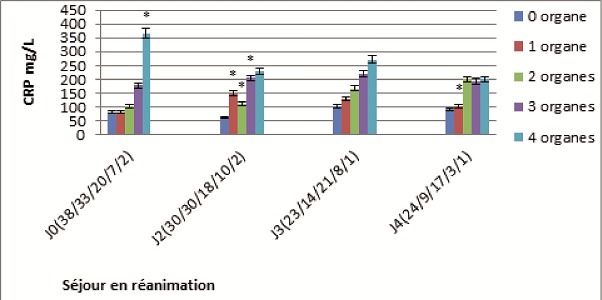
CRP en fonction de la défaillance multiviscérale définie par le score SOFA. Les chiffres entre parenthèse représentent le nombre de patients ayant 0, 1, 2, 3,4 organes défaillants +p < 0.05 vs 0 organe

L'analyse de la Figure 1 montre que les patients ayant un plus grand nombre d'organes défaillants ont la CRP la plus élevée: pour 4 organes défaillants et à l'admission (365 mg/L vs 80 mg/L, p < 0.05), pour 3 organes défaillants et au deuxième jour du séjour en réanimation (205 mg/L vs 60 mg/L, p < 0.05), pour 2 organes défaillants au deuxième jour du séjour en réanimation (110 mg/L vs 60 mg/L), pour 1 organe défaillant et au deuxième jour du séjour en réanimation (150 mg/L vs 60 mg/L). Les patients considérés comme n'ayant pas de défaillance d'organes (n = 38, 30, 23,24) sont ceux dont le SOFA est < à 3, des cas de traumatisme, de chirurgie d'urgence, d'hémorragie digestive haute et quelques cas de sepsis (Figure 1).

L'analyse du Tableau 2 nous montre que l'incidence de défaillance du système respiratoire est plus élevée chez les patients ayant une CRP> à 100 mg/L comparés à ceux ayant une CRP < à 10 mg/L à l'admission (groupe 3 vs groupe 1, p < 0,05) et au deuxième jour du séjour en réanimation (groupe 3 vs groupe 1, p < 0,05). L'incidence de survenue de la défaillance cardio-vasculaire est plus élevée chez les patients ayant une CRP > à 100 mg/L comparés à ceux ayant une CRP < à 100 mg/L au deuxième (groupe 3 vs groupe 1 et groupe 3 vs groupe 2, p < 0,05) et au troisième jour du séjour en réanimation (groupe 3 vs groupe 1 et groupe 3 vs groupe 2, p < 0,05) (Tableau 2).

**Tableau 2:** Relation entre les défaillances d'organe (SOFA 3 ou 4) et la CRP

Variables		Groupe 1 CRP < 10 mg/L	Groupe 2 CRP 10-100 mg/L	Groupe 3 CRP > 100 mg/L
CRP, n	J 0/2/3	35	32	33
Système respiratoire (%)	0	8 (23)	14 (44)	17 (51,5) †
	2	6 (20)	9 (32)	17 (53) †
	3	6 (32)	7 (39)	16 (52)
Système cardiovasculaire (%)	0	5 (14)	6 (19)	11 (33)
	2	6 (20)	7 (25)	20(62,5) †‡
	3	3 (16)	4 (22)	18 (58) †‡
Coagulation(%)	0	1 (3)	2 (6)	3 (9)
	2	2 (7)	2 (7)	3 (9)
	3	1 (5)	3 (17)	4 (13)
Foie (%)	0	0 (0)	0 (0)	2 (6)
	2	0 (0)	0 (0)	2 (6)
	3	0 (0)	0 (0)	2 (6,5)
Système nerveux (%)	0	9 (26)	9 (28)	9 (27)
	2	10 (33)	7 (25)	8 (25)
	3	5 (26)	3 (17)	10 (32)
Système rénal (%)	0	1 (3)	1 (3)	3 (9)
	2	1 (3)	1 (4)	3 (9)
	3	1 (5)	1 (6)	1 (3)

† p < 0,05 vs groupe 1

‡ p < 0,05 vs groupe 2

**Figure 2:**
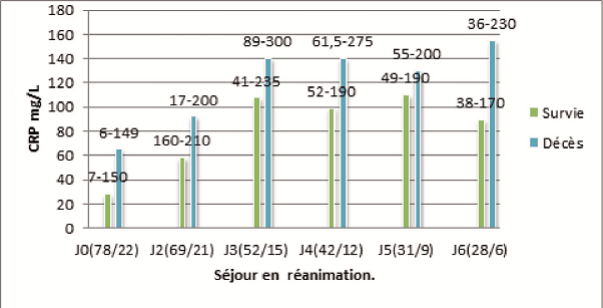
Evolution du taux de concentration de la CRP dans le temps durant le séjour en réanimation chez les vivants et chez les morts. Les chiffres entre parenthèse représentent les effectifs des vivants et des morts à J0, J2, J3, J4, J5, J6

Il ressort de la Figure 2 que chez tous les patients, vivants ou morts, la CRP s’élève pendant les premières 72 heures qui suivent l'admission en réanimation. A partir de la 72ème heure, le taux de CRP a tendance à baisser chez les survivants, par contre ce taux reste élevé et même a tendance à monter chez les morts (NS). A l'admission ou quel que soit le jour de l'hospitalisation en réanimation, la CRP est plus élevée chez des patients décédés que chez les vivants (NS). La mortalité globale est de 22% (Figure 2).

**Figure 3:**
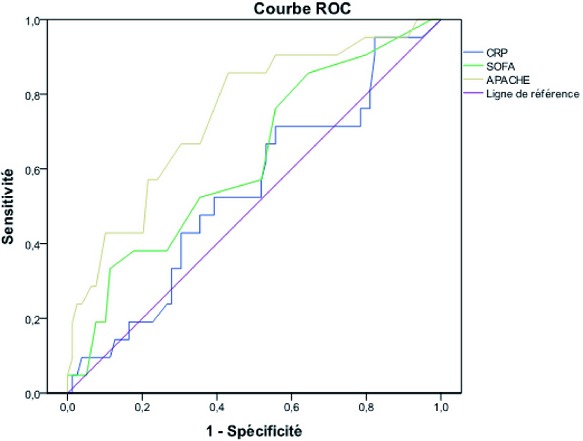
Courbe ROC du score APACHE II, du score SOFA et de la CRP dans les premières 24 heures

L'analyse du Tableau 3 montre que les ROC AUC des scores APACHE II, SOFA pour la mortalité à l'admission, à la 48ème heure et à la 72ème heure du séjour en réanimation sont respectivement plus élevées que celle de la CRP (Tableau 3). La Figure 3 montre que dans les 24 premières heures le score APACHE II est plus performant que le score SOFA total à l'admission qui est plus performant que le dosage de la CRP dans cette population d’étude (Figure 3).

**Tableau 3:** ROC AUC des différents scores pour la mortalité

	J0		J2		J3	
	ARE (95%CI)	p-value	AIRE (95%CI)	p-value	AIRE (95%CI)	p-value
APACHE	0.76 (0.65-0.88)	<0,001	0.76 (0.63-0.88)	<0.001	0.65 (0.48-0.82)	0.076
SOFA TOTAL	0.65 (0.51-0.78)	0 .038	0.76 (0.6-0.89)	<0.01	0.75 (0.62-0.88)	0.04
CRP	0.55 (0.42-0.69)	0.45	0.49 (0.35-0.64)	0.9	0.60 (0.43-0.75)	0.28

## Discussion

Nous avons eu environ un tiers seulement de malades qui avaient une CRP normale, un autre tiers avaient une valeur modérément élevée et le troisième tiers une valeur très élevée. Lobo et al. (2003) avaient à peu près la même répartition [14].

La CRP est directement associée à la sévérité de l'affection, reflétée par le score APACHE. Presterl et al. en 1997 ont aussi rapporté une association entre le score APACHE III et le score de sepsis MPII, et le niveau plasmatique de l'IL-6, TNF-Sr et la CRP. Dans leur étude, l'IL-6 et la CRP étaient tous les deux plus élevés chez les morts que chez les survivants. Pour les patients atteints de sepsis, ces auteurs ont préconisé d'inclure ces tests dans les scores de prédiction de la mortalité du sepsis et de la durée de séjour en réanimation. L'IL-6 coûte cependant plus cher que la CRP [15]. Des dosages d'interleukine 6 (IL-6) peuvent être intéressants, mais les variations au cours du temps sont parfois énormes et difficilement interprétables [17]. De même, le dosage de la procalcitonine (PCT) est plus coûteux que celui de la CRP [17] et son dosage n'est pas fait en routine aux cliniques universitaires de Bruxelles.

Comme attendu, le pourcentage des patients présentant une infection était également plus élevé chez les patients ayant un taux de CRP > 10 mg/L que chez ceux ayant un taux de CRP <10mg/L. En effet, la CRP est une protéine qui permet de faire le diagnostic et de suivre l’évolution des phénomènes inflammatoires. La CRP est élevée dans le sepsis [17–23]. La majeure fonction de la CRP est de fixer la phosphocholine qui permet la reconnaissance des pathogènes et des phospholipides provenant de la destruction cellulaire [24]. Notons cependant que la PCT, quant à elle, est un excellent marqueur reflétant la présence ou la sévérité d'une infection bactérienne [25–27]. La PCT a une supériorité documentée par rapport aux marqueurs conventionnels tel que la CRP ou les globules blancs, qui ont perdu l'exactitude diagnostique et induisent parfois en erreur; la PCT a un rôle significatif en facilitant le diagnostic précoce et la prise en charge de l´infection bactérienne et baisse ainsi la morbidité et la mortalité y associées [27, 28].

Les patients ayant une CRP élevée sont plus longtemps hospitalisés en réanimation et ont une mortalité plus élevée. Ainsi De Beaux et al. en 1996 observent, lors de la mesure des concentrations des médiateurs inflammatoires dans la pancréatite aigüe, qu'une élévation précoce et persistante (à la 48ème heure) de la CRP est prédictive d'un long séjour à l'hôpital et d'une augmentation de la mortalité en cas de pancréatite aiguë [9]. Nous avons également trouvé qu'il y a une association entre l’élévation de la CRP, du score SOFA à l'admission et du score SOFA maximum. Les patients ayant un plus grand nombre d'organes défaillants ont la CRP la plus élevée.

Dans notre étude nous avons trouvé que la CRP est corrélée à l'incidence de la défaillance du système respiratoire et du système cardiovasculaire. Il n'y a par contre pas de corrélation statistiquement significative entre l’élévation de la CRP et la défaillance des autres organes. Cela serait dû à la taille de l’échantillon. En effet, alors qu'il y a plus de patients avec une défaillance du système respiratoire et cardiovasculaire, il y a peu de patients présentant les autres défaillances. Il serait intéressant dans l'avenir d'inclure plus de patients afin de voir si cette corrélation serait statistiquement significative.

A l'admission nous avons eu 38 patients qui n'avaient pas de défaillance d'organes. Cela serait dû au fait que nous avons considéré une défaillance d'organe pour un score SOFA égale à 3 ou à 4 et que certains patients avaient été admis en réanimation pour traumatisme, chirurgie d'urgence, hémorragie digestive haute et sepsis.

Nous avons également trouvé que la mortalité globale était de 22% et les morts avaient un taux de CRP plus élevé que les survivants à l'admission, dans les 48 heures et les 72 h du séjour en réanimation. A l'admission ou quel que soit le jour de l'hospitalisation en réanimation, la CRP est plus élevée chez les morts que chez les vivants. A partir de la 72ème heure du séjour en réanimation le taux de CRP a tendance à baisser chez les survivants, par contre ce taux reste élevé et même a tendance à monter chez les morts. Notons qu'il s'agit ici d'une tendance mais cette corrélation n'est pas statistiquement significative (Figure 2).

L’étude réalisée par Lobo et al. a démontré que le taux sérique de la CRP est un bon marqueur de la mortalité pour les patients souffrant d'une défaillance multi viscérale. La CRP associée au score APACHE II permettait de prédire les patients à risque de décès. Ils ont trouvé qu'il y avait plus de décès dans le groupe où la CRP était plus élevée [14]. Plusieurs mesures de la CRP permettaient d'identifier les patients nécessitant un diagnostic, un suivi et un traitement plus soutenus pour éviter les complications. La répétition de la CRP permet de suivre si le patient répond au traitement. Elle permettrait aussi dans les essais cliniques de déterminer les patients à haut risque pouvant bénéficier de nouvelles interventions thérapeutiques. Lobo et al. avaient beaucoup plus de patients dans leur étude que dans la nôtre. Il serait intéressant dans l'avenir de faire une étude avec un nombre de patients semblable ou supérieur à celle de l’étude de Lobo et al. afin de voir si la CRP reste toujours corrélée à la mortalité.

Quant à elle, la PCT fait l'objet d'un intérêt croissant dans l'arbre décisionnel pour l'antibiothérapie. En effet, une étude réalisée randomisée contrôlée réalisée par Schuetz et coll. montre une baisse significative de la prescription d'antibiotiques, de la durée de l'antibiothérapie et de l'apparition d'effets indésirables à 30 jours. Mais ces résultats doivent encore être confirmés et validés par d'autres études [29]. Presterl et al. avaient quant à eux trouvé que la CRP était significativement élevée chez les morts que chez les vivants. Dans leur étude, chez les patients en choc septique, la CRP s’élevait du 3ème au 7ème jour [18].

Notons cependant que l'utilité de la CRP est sujette à controverses. Ainsi par exemple, Bauer et al. (2010) dans leur étude sur la pneumonie acquise dans la communauté (PAC) stipulent que de nombreuses études s'intéressent à l'utilité de la CRP, pour évaluer le pronostic des patients mais que six études sur dix ne trouvent pas d'associations entre la valeur de la CRP à l'admission et la mortalité à 30 jours et 5 études sur 8 ne trouvent pas d'association entre la valeur de la CRP à l'admission et les scores d’évaluation pronostique et que les revues de la littérature parlent contre une association entre la CRP et la valeur pronostique. Par rapport à l'utilité dans le suivi, 8 études sur 10 retrouvent une association entre la CRP et l’échec de traitement [30]. Et dans une analyse rétrospective des valeurs de la CRP obtenues à l'admission chez 351 patients hospitalisés dans un centre hospitalier universitaire, les auteurs ne trouvent à la CRP ni une valeur diagnostique, ni une valeur pronostique pour les critères tels que la mortalité, l'admission en réanimation, la durée de séjour et le retour à domicile [30]. La conclusion de Bauer et al. est que l'utilité de la CRP pour la prise en charge de la PAC reste largement controversée et qu'ils constatent un décalage important entre la perception que les médecins ont quant à l'utilité de la CRP et déconseillent le recours systématique à la CRP pour le diagnostic, le pronostic et le suivi des patients avec une PAC [30].

Dans cette étude nous avons cherché à déterminer si le la CRP est un bon marqueur de gravité chez les patients admis en réanimation. Nous n'avions pas l'objectif de chercher le pouvoir discriminant de la CRP sur la présence ou non de certaines pathologies. Ainsi par exemple, J. Lemarié et al. en parlant de l'analyse de la performance diagnostique d'un biomarqueur stipule que pour le domaine du sepsis par exemple, l’évaluation de la sensibilité ou de la spécificité d'un biomarqueur quelconque n'a que peu d'intérêt clinique: ces deux paramètres sont définis dans une population où le statut septique et non septique est déjà connu, et de ce fait ne correspondent pas à la question que se pose le clinicien dans sa démarche diagnostique [31].

Les courbes ROC permettent de choisir le seuil qui permettra de combiner les meilleures sensibilités et spécificités en fonction de ce que l'on veut privilégier. L'aire sous la courbe ROC (AUC) est équivalente à la probabilité pour le biomarqueur d'avoir une valeur plus élevée pour un malade que pour un non-malade et évalue donc son pouvoir discriminant. Mais les courbes ROC ne sont pas un bon reflet de la valeur ajoutée d'un biomarqueur, surtout lorsque l'AUC du modèle de prédiction clinique est déjà élevée [32].

Il y a plusieurs limitations à cette étude: Bien que la prévalence des patients présentant une infection soit plus élevée dans les groupe 2 et 3 que dans le groupe 1, les patients n'ont pas été séparés dans deux sous-groupes avec ou sans infection. En effet, la taille de l’échantillon ne permet pas d'autres subdivisions intéressantes, telles que patients avec infection versus patients sans infection, qui réduirait encore considérablement la population de l’étude. Il serait plus judicieux ultérieurement d'effectuer l’étude sur une population plus importante de patients et de de voir la contribution de la gravité ou du sepsis dans l’élévation de la CRP.

Il faudra aussi ultérieurement voir l´intérêt de la CRP en plus des scores classiques (APACHE ou SOFA) en réalisant une étude multivariée sur une population plus importante de patients pour vérifier si la CRP est un facteur indépendant de mortalité et, si oui, comparer la valeur discriminante de chacun de ces facteurs évaluée par des courbes ROC. Il serait intéressant de vérifier si la CRP permettrait d´améliorer la valeur prédictive des scores APACHE II et SOFA pour la mortalité.

Bien que n’étant pas l'objectif de ce travail, il serait intéressant aussi ultérieurement d'une part d’étudier les aspects épidémiologiques liés aux infections: leur prévalence, leur incidence, leur étiologie confirmée ou non par la microbiologie, le type d'infections et d'autre part d'effectuer une étude multicentrique ainsi que l’évaluation d´autres biomarqueurs tel que la procalcitonine, l'endocan, le supar, l'albumine…

## Conclusion

Les scores de gravité (score APACHE et score SOFA à l'admission) sont significativement plus élevés chez les patients présentant un taux sérique de CRP > 100mg/L comparés à ceux qui ont un taux < 10 mg/L. Le score SOFA max et la durée du séjour en réanimation sont significativement plus élevés chez les patients présentant un taux sérique de CRP > 100mg/L comparés à ceux qui ont un taux < 100 mg/L. Le pourcentage de patients présentant une infection est plus élevé chez les patients ayant un taux sérique de CRP > 10mg/L comparé à ceux ayant un taux sérique < 10 mg/L.
